# Implication of integrins α3β1 and α5β1 in invasion and anoikis of SK-Mel-147 human melanoma cells: non-canonical functions of protein kinase Akt

**DOI:** 10.18632/aging.202243

**Published:** 2020-12-01

**Authors:** Nadezhda I. Kozlova, Galina E. Morozevich, Nina M. Gevorkian, Albert E. Berman

**Affiliations:** 1 VN Orekhovich Institute of Biomedical Chemistry, Moscow 119121, Russia

**Keywords:** integrins, tumor invasion, anoikis, proteinkinases

## Abstract

Downregulation of integrins α3β1 and α5β1 strongly decreased cell colony formation and in vitro invasion and markedly enhanced anoikis in SK-Mel-147 human melanoma cells. These modifications were accompanied by a marked increase in the levels of active Akt protein kinase, which indicated it played a non-canonical function in the melanoma cells. Pharmacological inhibition of Akt1, an Akt isozyme, in cells depleted of α3β1 or α5β1 restored their invasive activity, while inhibition of the Akt 2 isoform did not cause a visible effect. Similar to our previous results with the α2β1 integrin, this finding suggested that in signaling pathways initiated by α3β1 and α5β1, the Akt1 isoform performs a non-canonical function in regulating invasive phenotype of melanoma cells. In contrast, when the effects of Akt inhibitors on anoikis of the melanoma cells were compared, the Akt2 isoform demonstrated a non-canonical activity in which Akt2 suppression led to a significant attenuation of apoptosis in cells with downregulated α3β1 or α5β1. Our results were the first evidence that, in the same tumor cells, different integrins can control various manifestations of tumor progression through distinct signaling pathways that are both common to various integrins and specific to a particular receptor.

## INTRODUCTION

A hallmark of tumor progression is the ability of tumor cells to survive and grow in the absence of contact with the extracellular matrix (substrate). Generally, normal cells that lack communication with the matrix die by a mechanism called anchorage-dependent apoptosis, or anoikis; tumor cells have a mechanism for overcoming anoikis. Another phenotypic feature of tumor progression is the ability of tumor cells to penetrate and destroy surrounding tissues through invasion, and form local and distant metastases. Both of these features result from significant modifications in matrix–cell interactions or changes in direct and reverse signal flows between the cells and the matrix [1, 2].

Integrins are major signal mediators in matrix–cell interactions. Research has shown that they play a key role in tumor growth and progression [3–6]. The integrin family has 24 members, each of which is a heterodimer consisting of an α and a β chain linked by non-covalent bonds. Integrin receptors differ in ligand specificity and expression level in mammalian tissues. The most common and vital integrins for cells are the fibronectin-binding integrin (α5β1), the collagen-binding receptor (α2β1), and the laminin-binding integrin (α3β1). These integrins are the subject of a majority of studies aimed at elucidating the role of integrin-mediated signaling in the growth and progression of tumors [7–9]. However, studies on individual integrins have shown contradictory findings. This might be due to the diversity of the integrin family. Studies on α5β1 [9–12] and α3β1 [13–15] have also concluded that a variety of signaling pathways can induce the same receptors in different cells.

The effect of integrins on cell behavior is carried out indirectly through the signaling pathways they initiate. However, data on the signaling mechanisms underlying the effects of these receptors on tumor phenotype remain largely unclear.

For example, in immortalized mouse keratinocytes, the stimulatory effect of α3β1 on invasion is realized via the extracellular signal-regulated kinase (Erk)-dependent signaling pathway [16]. The involvement of α3β1 in promotion of the aggressive phenotype of glioblastoma cells is mediated through the activation of FAK and the small GTPases Cdc42 and Rac1 [17]. An investigation on a breast carcinoma line showed that α5β1 supports cell resistance to anoikis by activating the MAP kinase pathway [7], while another study on bone marrow stem cells indicated that the blocking effect of α5β1 on anoikis was caused by increased levels of nitric oxide [18]. In contrast, researchers into gastric cancer cells found that an increase in α5β1 expression stimulated anoikis, which was attributed to an increase in ROS production [12]. Thus, the effects exerted by the integrins on tumor progression may depend on differences in the signaling pathways initiated by a specific integrin in different cell types. However, because all types of normal cells and tumor cells express a number of different integrins on their cell membrane, the cumulative effect may depend on the contribution of each integrin. Therefore, it is important to study the signaling pathways of various receptors in a particular cell type.

We have previously shown that α2β1 controls the invasion and anoikis of human SK-Mel-147 melanoma cells through a mechanism based on the non-canonical activity of Akt1, one of three Akt protein kinase isozymes, which demonstrated its function in suppressing invasion and promoting anoikis in melanoma cells [19, 20]. In this study, we looked at the characteristics of signaling pathways initiated by α3β1 and α5β1, as well as their roles in the mechanisms of invasion and anoikis in melanoma cells. We showed that the effects of α3β1 and α5β1 are mediated through the non-canonical activity of Akt1 and Akt2 isoforms. In tumor cells, different integrins can control various manifestations of tumor progression through distinct signaling pathways that are both common to various integrins and specific to a particular receptor.

## RESULTS AND DISCUSSION

### Downregulation of the expression of α3β1 and α5β1 inhibits colony formation and invasive activity of melanoma cells and reduces the resistance of the cells to anoikis

**Figure 1:**
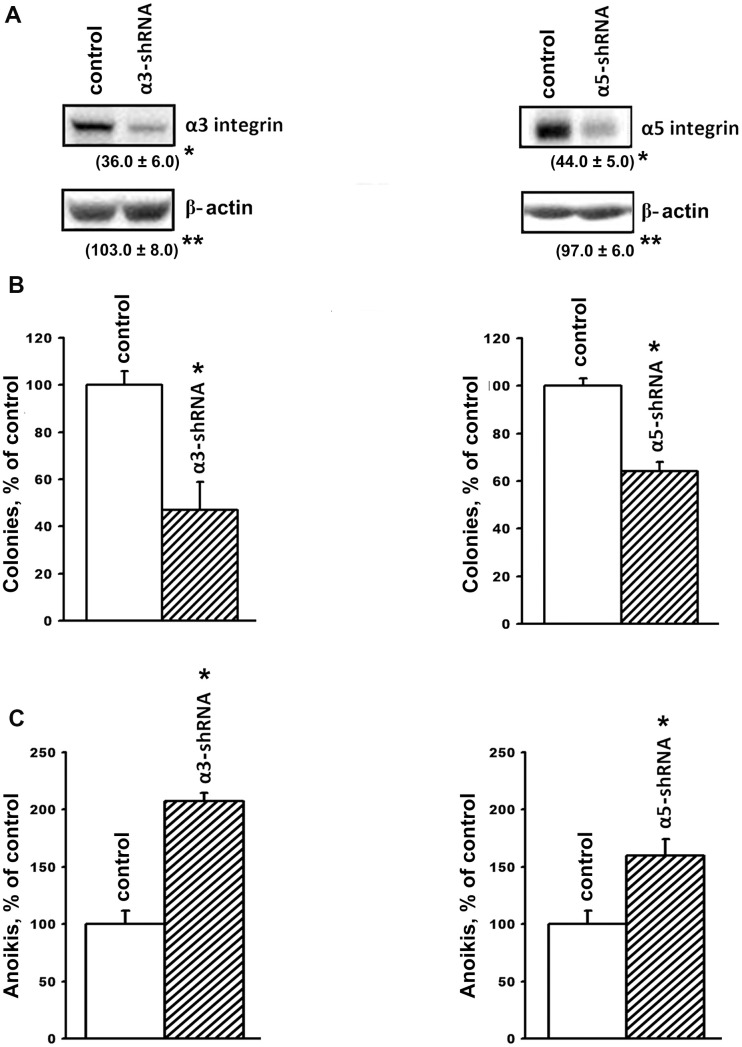
**Downregulation of the expression of α3β1 and α5β1 integrins reduces clonal activity and enhances anoikis of SK-Mel-147 cells.** The cells were transduced with a lentiviral plasmid vector pLKO.1-puro containing α3- or α5- shRNA or with the control (scrambled shRNA) vector and selected using puromycin. (**A**) Western-blotting of the cellular lysate proteins. Cell lysate proteins were run on SDS-PAGE, western-blotted and probed as described in Materials and Methods. The blots were probed with 1:1000 dilution of antibodies to the specified proteins and treated as described in Materials and Methods. Shown are representative blots. Numbers below the bands indicate the ratio (%) protein level in integrin shRNA transfected cells compared to control shRNA transfected cells normalized against β-actin. Results of three independent experiments are shown (M ± SEM). *ρ < 0.05, **non-significant. (**B**) Effect of α3β1 or α5β1 downregulation on clonal capacity of the cells. Cells transduced with the scrambled or α3/α5- shRNA-containing vectors were treated as described in Materials and Methods; results of three independent experiments are shown (M ± SEM); *p < 0.01, relative to control. (**C**) Effect of the downregulation of α3β1 or α5β1 on anoikis of the cells. Cells were transduced with the scrambled (PLKO.1) or α3- or α5- shRNA-containing vectors and cultured on poly-HEMA as described in Materials and Methods.

In this study, integrin expression was downregulated by transducing the cells with plasmid clones expressing α3- or α5-specific shRNAs. A Western blot analysis showed a high efficiency of these clones; the expression of α3β1 or α5β1 in the SK-Mel-147 cells transduced with α3 shRNA or α5 shRNA was much lower than in the control cells (Figure 1A).

Tumor progression is linked with the ability of tumor cells to grow in the absence of contact with the extracellular matrix and to form colonies in semi-liquid media, such as agarose and methyl cellulose. The ability of tumor cells to overcome anoikis is largely determined by the cell surface molecules, which include integrins. We previously demonstrated that blocking the expression of α2β1 in the SK-Mel-147 line led to a 2-fold decrease in the clonal activity of melanoma cells [19]. In this investigation, we found that suppression of α3β1 resulted in approximately the same decrease in the clonal activity of these cells (Figure 1B). The role of α3β1 and α5β1 in maintaining the viability of SK-Mel-147 cells in the absence of matrix–cell contact was confirmed by studying their growth on a non-adhesive substrate (Figure 1C). The suppression of α3β1 and α5β1 increased anoikis by 2.0 and 1.6 times, respectively, which clearly correlates with the results shown in Figure 1B. We previously observed a similar effect caused by the suppression of α2β1 in the SK-Mel-147 cells [19]. Thus, we found that the three integrins with different ligand specificity stimulated the clonal activity of melanoma cells and increased their resistance to anoikis.

**Figure 2:**
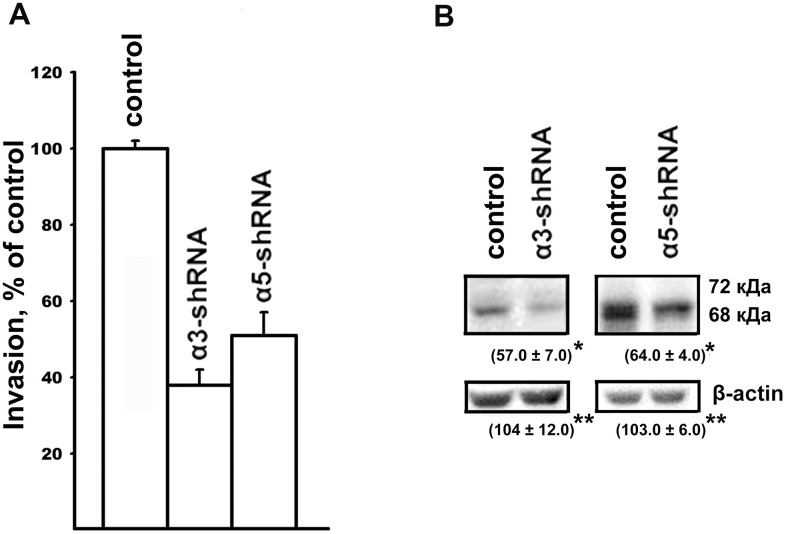
Effect of α3β1 or α5β1 on *in vitro* invasion (**A**) and the level of MMP-2 collagenase (**B**) in SK-Mel-147 cells. (**A**) and the level of MMP-2 collagenase (**B**) in SK-Mel-147 cells. (**A**) The cells were transduced with the control or α3/α5 shRNA vectors, applied on matrigel and treated as described in Materials and Methods. The number of migrated cells transduced with the control vector was taken as 100%. Results of four independent experiments are shown (M ± SEM). (**B**) Cell lysate proteins were run on SDS-PAGE and western-blotted as described in Materials and Methods. The blots were probed with 1:300 dilution of MMP-2 antibodies and treated as described in Materials and Methods. Shown are representative blots. Numbers below the bands indicate the ratio (%) protein level in integrin shRNA transfected cells compared to control shRNA transfected cells normalized against β-actin. Results of three independent experiments are shown (M ± SEM). *ρ < 0.05, **non-significant.

Along with resistance to anoikis, tumor progression is associated with an increase in the invasive activity of tumor cells. In this study, implication of α3β1 and α5β1 in the mechanisms of tumor cell invasion was evaluated by downregulating the receptors and looking at the effect on the *in vitro* invasion and levels of matrix metalloproteinases (MMPs) by the SK-Mel-147 cells. In a previous study, we showed that the suppression of α2β1 led to a 2-fold decrease in the *in vitro* invasion and a significant decrease in the activity of collagenases MMP-2 and MMP-9 in melanoma cells [19]. As can be seen from the data presented in Figure 2, blocking the expression of the α3β1 and α5β1 receptors was accompanied by a sharp decrease in the *in vitro* invasion and in the level of the active (68 kDa) form of collagenase MMP-2 in melanoma cells. Thus, as with the clonal activity, the three integrins differing in ligand specificity were found to have a stimulating effect on the *in vitro* invasion of melanoma cells. In contrast with the results presented in the cited article [19], collagenase MMP-9 and the inactive (72 kDa) form of MMP-2 were not found in this study. This was likely due to the use of cell cultures differing in the number of cell doublings.

The data found in this study agreed with the results of other studies that used culture models of tumors with different origins. For example, one study found that the downregulation of α3β1 in breast carcinoma cells with high metastatic potency led to a decrease in their *in vitro* invasion and tumorigenic activity [13]. Another study found that mouse keratinocytes, expressing E7 oncoprotein acquired invasive properties only if α3β1 was expressed on the surface of the cells [14]. Sharp increases in the *in vitro* invasions of colon carcinoma and gastric cancer cells occurred upon activation of some transcription factors, was shown to be due to the increased expression of α5β1 [9]. The data found in this investigation, using melanoma cells, as well as the results of our previous studies on breast carcinoma cells [21] showed that the knock-down of α5β1 significantly reduced the invasive activity of the cells and suppressed the activity of collagenase MMP-2. Our results demonstrated the protective effect of α3β1 and α5β1 against anoikis in melanoma cells and were confirmed by studies on cells of various origins, such as gastric cancer cells [22], primary prostate epithelium cell cultures [23], and bone marrow stem cells [18].

### Signaling pathways that mediate effects caused by inhibition of α3β1 and α5β1: non-canonical functions of Akt1 and Akt2 in anoikis and invasion of SK-Mel-147 cells

**Figure 3:**
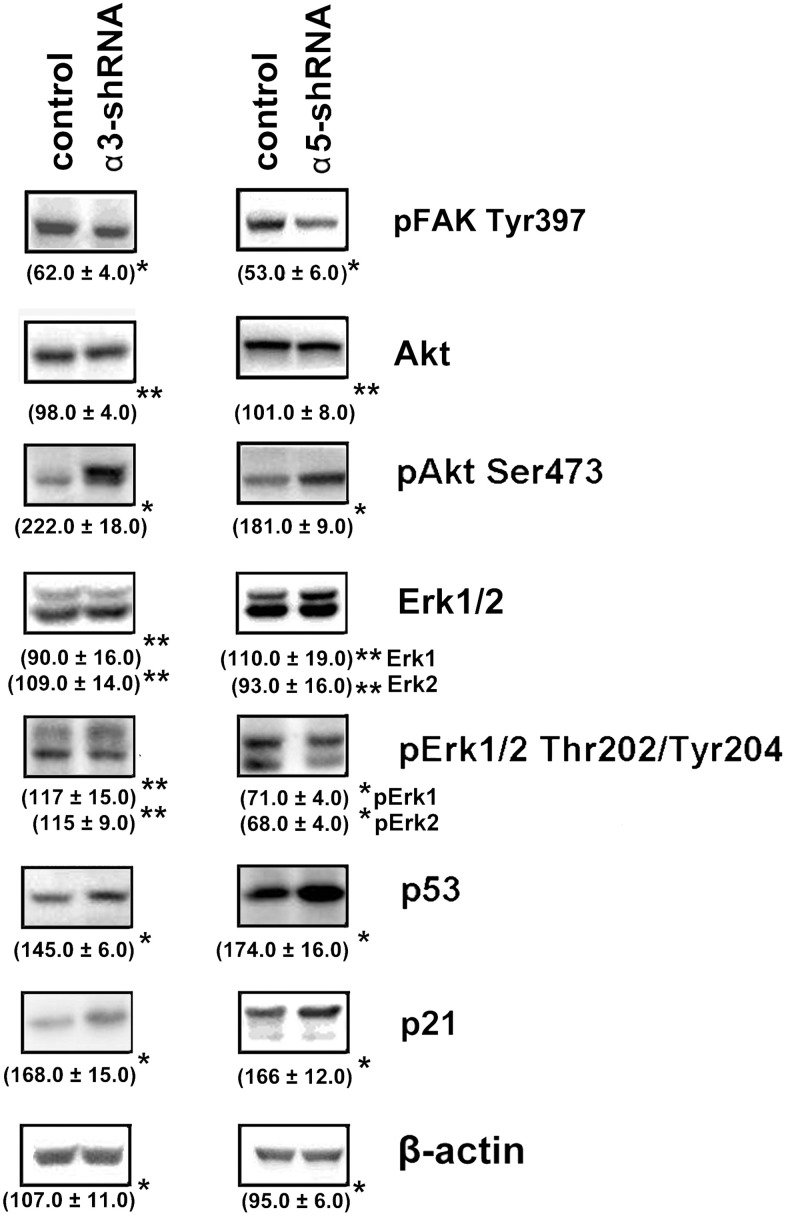
**Effect of knock-down of α3β1 or α5β1 on expression of signaling proteins in SK-Mel-147 cells.** The cells were transduced with the scrambled shRNA or α3/α5 shRNAs, cell lysate proteins were run on SDS-PAGE and western-blotted as described in Materials and Methods. The blots were probed with 1:1000 dilution of antibodies to the specified proteins. Shown are representative blots. Numbers below the bands indicate the ratio (%) protein level in integrin shRNA transfected cells compared to control shRNA transfected cells normalized against β-actin. Results of three independent experiments are shown (M ± SEM). *ρ < 0.05, **non-significant.

To clarify the mechanisms underlying the impact of α3β1 and α5β1 on tumor phenotype, we analyzed the expression of the signaling proteins that have been implicated in regulating various physiological functions of cells. A western blot analysis of cell protein lysates showed that blocking the expression of the two integrins led to a sharp increase in the levels of the apoptogenic protein p53 and the cell cycle inhibitor protein p21 (Figure 3). Many studies have demonstrated that these proteins inhibit tumor growth [24, 25]. The levels of p21 and p53 increased during suppression of invasion and stimulation of anoikis induced by downregulating α3β1 and α5β1, which indicated that p21 and p53 were involved in the signaling of these receptors. We also analyzed the levels of FAK, Erk, and Akt, which are other signaling proteins that play a role in integrin-dependent signaling. The activity of these enzymes was evaluated by determining the levels of their phosphorylated (active) forms in the cell lysates. As can be seen from Figure 3, the suppression of each of the receptors was accompanied by significantly reduced levels of FAK. FAK mediates the involvement of integrins in the signaling pathways, which determines the motility of the tumor cells, stimulates production of collagenases MMP-2 and MMP-9, and enhances the invasive activity [26]. Thus, a decrease in the level of the active form of FAK in cells with downregulated α3β1 and α5β1 receptors correlated with a reduction in the invasive activity of the cells and these results suggested that FAK was involved in signaling of both integrins. Figure 3 also shows that the suppression of α3β1 did not affect the levels of the total Erk kinase isomers (42 kDa and 44 kDa) and their phosphorylated forms in SK-Mel-147 cells. However, blocking α5β1 caused a noticeable decrease in the levels of the active forms, although it did not affect the content of the total protein. Erk is the final link in the Ras/Raf/MEK/Erk cascade that transfers integrin- or growth factor-initiated signals controlling cell proliferation, apoptosis, and cell senescence [27]. In a particular cell population, the choice of any of these responses depends on the intracellular localization of Erk and the strength of its signal (i.e., the level and duration of its active state) [28]. One would suggest that α3β1 and α5β1 differ in the strength of the Erk signal they induce in melanoma cells and α3β1 controls invasion and anoikis in these cells via Erk-independent pathways.

**Figure 4:**
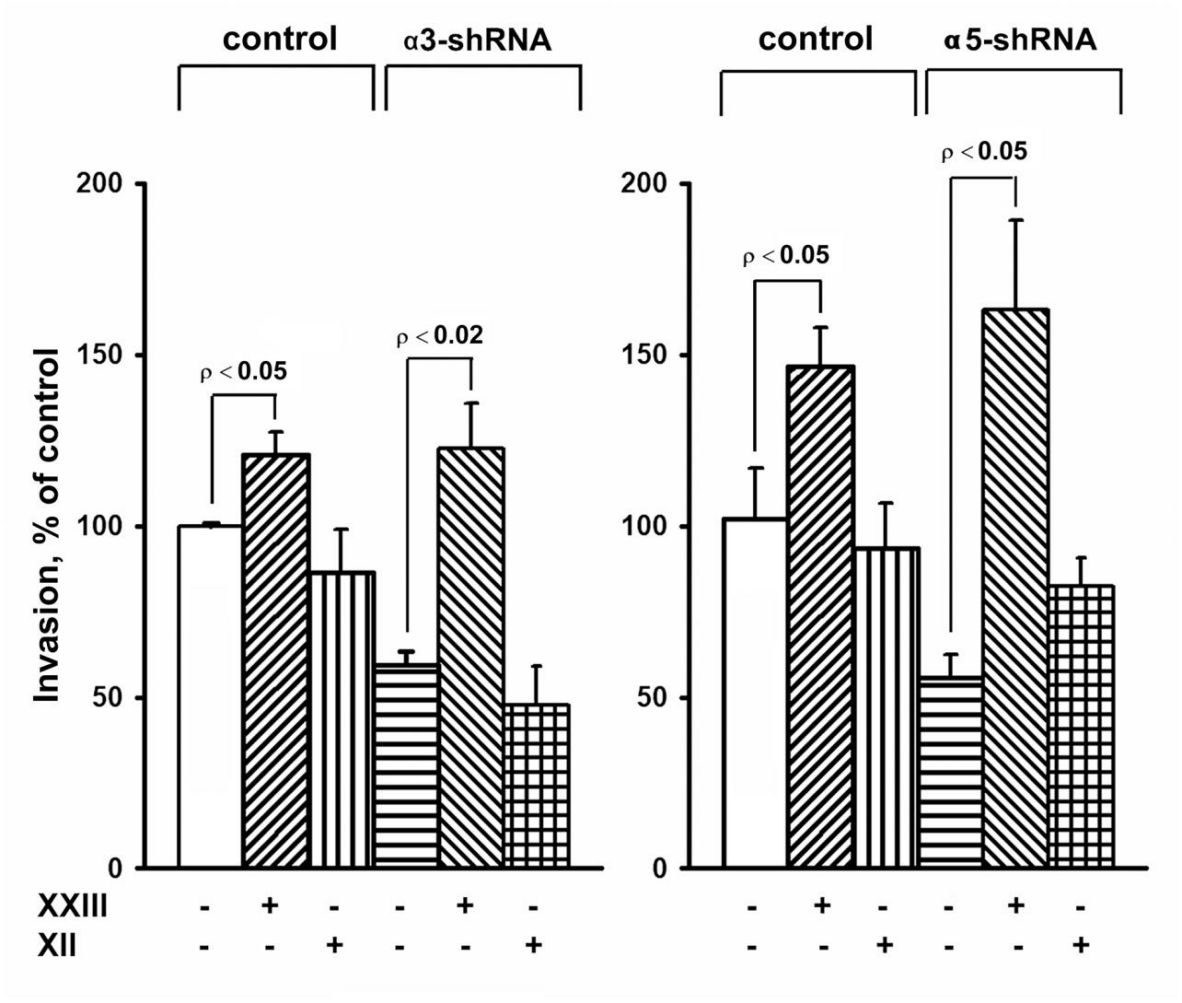
**Effect of Akt isoform inhibitors on *in vitro* invasion of SK-Mel-147 cells.** Cells transduced with an appropriate shRNA were incubated 48 hours with 3 μM Akt1-specific inhibitor XXIII or 5 μM Akt2-specific inhibitor XII, after which the *in vitro* invasion was determined as described in Materials and Methods. The invasion of cells transduced with a control vector and not treated with inhibitors was taken as 100%. The results of 3 independent experiments (M ± SEM) are presented.

**Figure 5:**
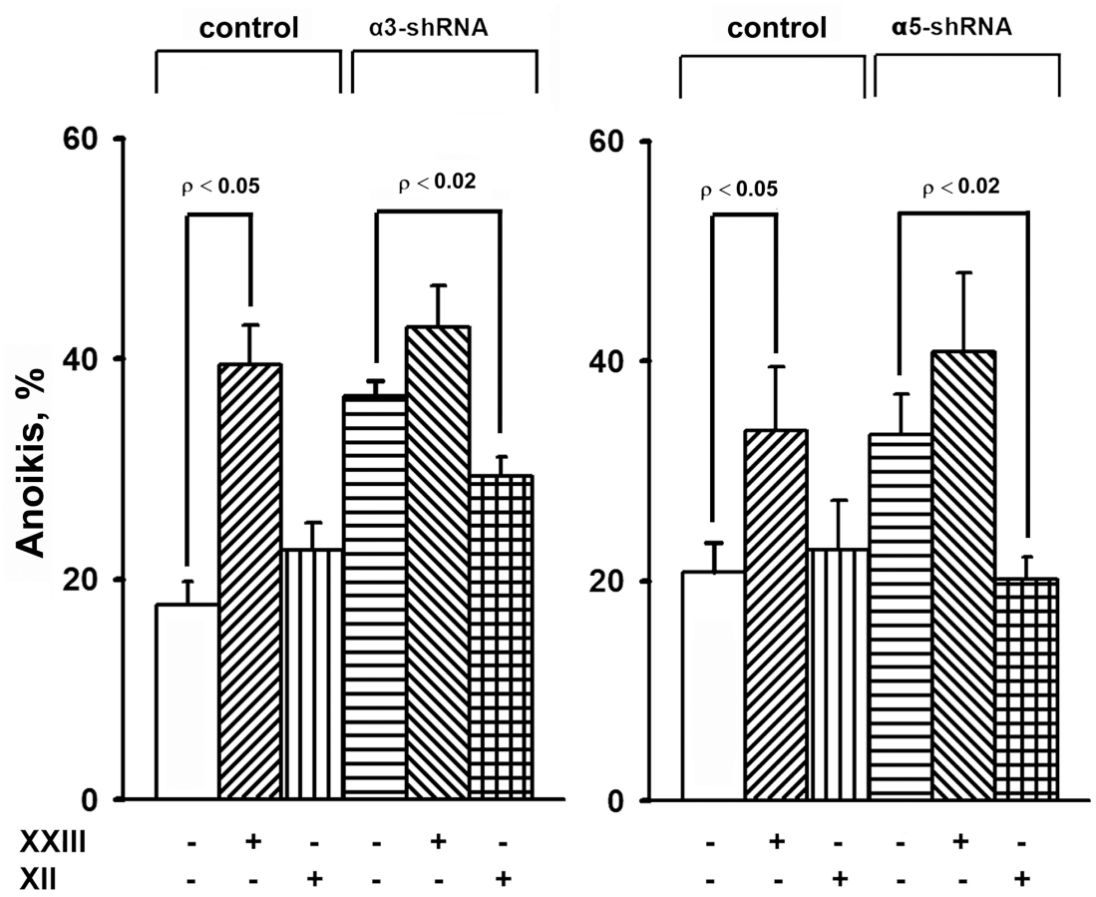
**Effect of Akt isoform inhibitors on anoikis of SK-Mel-147 cells.** Cells transduced with control or α3/α5 shRNA were cultured on poly-HEMA as described in Materials and Methods. Prior to applying on poly-HEMA the cells were treated for 24 h at 37° C with 3 μM Akt1-specific inhibitor XXIII or 5 μM Akt2-specific inhibitor XII. Anoikis was determined from percentage of Trypan Blue-stained cells. The results of four independent experiments are shown (M ± SEM).

The results of this study also showed changes in the Akt protein kinase activity (Figure 3). As shown in the figure, there is a significant increase in the expression of the active form of Akt in response to the knock-down of the investigated integrins in melanoma cells. Most studies on Akt in oncogenesis have indicated that it plays a key role in the mechanisms involved in the invasion of tumor cells and their resistance to apoptosis. [29]. Therefore, an increase in the level of its active form in cells with reduced invasive activity and increased sensitivity to anoikis could indicate that Akt performs an unusual, or non-canonical, function in suppressing invasion and stimulating anoikis. In our recent studies, we showed that Akt1 protein kinase, one of three isoforms of the Akt family, suppressed an *in vitro* invasion and enforced anoikis in SK-Mel-147 cells depleted of α2β1 [19, 20]. We were interested in determining whether a similar mechanism was realized in the same cells upon suppression of α3β1 and α5β1, and whether Akt1 and/or another Akt isoform was involved in the mechanism. In this study, we used antibodies to phospho-Akt that were not isozyme-specific. Thus, an increase in its level would only be a trait that accompanied, but was not related to, the changes in invasion and anoikis of melanoma cells. To this end, the effect of isoform-specific Akt inhibitors on *in vitro* invasion and anoikis of SK-Mel-147 cells was analyzed (Figures 4, 5). Figure 4 shows that the inhibition of the Akt2 isoform did not significantly affect the *in vitro* invasion of the control cells and the cells with a reduced expression of each integrin. In contrast, inhibition of Akt1 markedly enhanced *in vitro* invasion of the control cells and greatly increased the invasive activity of the cells with a downregulated expression of both integrins.

When determining the effect of inhibitors of Akt isoenzymes on anoikis of melanoma cells, we found that treatment with an Akt1 inhibitor significantly enhanced anoikis of the control cells (Figure 5). However, there was no visible impact on the anoikis of cells that had a reduced expression of α3β1 or α5β1. Cell treatment with an Akt2 inhibitor did not significantly affect anoikis of the control cells, whereas inhibition of Akt2 in cells depleted of α3β1 or α5β1 significantly reduced their anoikis (i.e. increased their resistance to this type of apoptosis).

The results of this investigation and previously obtained data [19, 20] showed that α2β1, α3β1, and α5β1, which differ in ligand specificity, have a common function: they enhance tumor progression of melanoma cells by stimulating the invasive phenotype and counteracting anoikis. However, the signaling mechanisms involved in these functions may be specific for both the individual receptors and phenotypic manifestations of progression. Each receptor controls the invasive phenotype of the SK-Mel-147 cells using a mechanism that involved the non-canonical functions of the Akt1 isoenzyme. In contrast, differences were found between the integrins in the signaling pathways controlling anoikis in these cells. For example, α2β1-dependent pathway was mediated via a non-canonical function of Akt1 [20], while α3β1- and α5β1-initiated pathways involved a non-canonical function of Akt2 (Figure 5).

Figure 5 shows that Akt2 exhibits noncanonical activity only in unattached cells depleted in integrins α3β1 or α5β1. In the unattached cells with unmodified expression of these receptors, Akt2, unlike Akt1, apparently does not transduce signals affecting anoikis, since its pharmacological suppression does not stimulate or suppress cell death. Similarly, Akt1 showed a non-canonical activity only in the unattached melanoma cells lacking integrin α2β1 [20]. A possible explanation for these transitions is a difference in intracellular localization of Akt isoforms. Akt2 was shown to localize at sites adjacent to the extracellular matrix and at the mitochondria while Akt1 was found mainly in cytoplasm [29, 30]. One would speculate that depletion of integrins in the cell membrane leads to relocation of Akt2 to another compartment followed by activating its noncanonical properties. This assumption is supported by the observation on another key signal kinase, Erk, which exhibits unusual (non-canonical) pro-apoptotic activity only upon its aberrant nuclear localization [27].

We have shown in our previous studies on human breast carcinoma and melanoma lines that downregulation of integrins α2β1 and α5β1 resulted in reduction of invasion, increase in anoikis and suppression of Erk activity, and similar phenotypic changes were observed in response to inhibition of this kinase [19–21, 31]. These findings support the involvement of Erk in α2β1- and α5β1- initiated pathways controlling invasion and anoikis in the cells in study. In the present investigation, α3β1 knock-down induced above changes in invasion and anoikis in SK-Mel-147 cells albeit did not affect activation of Erk (Figures 1, 3) A possible explanation is that in these cells, α2β1 and α5β1 control invasion and anoikis through Akt- and Erk-dependent signaling pathways, while α3β1 realizes this control through the AKT-dependent pathway. Published studies indicates that Akt and Erk can act independently of each other [32].

Numerous studies have described the non-canonical activities of Akt isoenzymes. The particular manifestations of the properties of Akt isoenzymes, such as the promotion or suppression of certain phenotypes, are cell type-specific. For example, one study on a prostate cancer line showed that Akt1 and Akt2 had a negative effect on *in vitro* invasion and cell migration [33]. However, a study on two lines of breast cancer cells found that Akt2 stimulated these activities, while Akt1 stimulated proliferation and inhibited invasion and migration [34]. Results of this study were the first to show that in the same cells, different integrins are able to control various manifestations of tumor progression through signaling pathways mediated by distinct Akt isoforms. These findings were obtained in a single melanoma cell line, so further investigations are required to clarify how widespread this phenomenon is among other tumor types.

The diverse mechanisms underlying the non-canonical activities of the Akt family are characterized by the cell type-specific patterns of the signaling pathways, which can interact with and oppose each other. For example, a study on a breast carcinoma line found that the anti-invasive and anti-migration activity of Akt1 is mediated via the actin-binding protein palladin, which is activated by Akt1 and not Akt2, followed by the aggregation of actin filaments and the blockage of the invadopodia formation [34]. However, in a prostate cancer cell line, an invasion suppressive mechanism was identified that was triggered by both Akt1 and Akt2 isoforms through different pathways. The Akt1 isoform inactivated the β1 integrins followed by interrupting the signal transfer to the EGFR and MET receptor tyrosine kinases, while the Akt2 isoform inhibited the synthesis of miR-200, thereby abrogating the miR-200-induced activation of the β1 integrins [33]. These data are consistent with our findings that demonstrate the role of Akt1 in the control of an invasive phenotype of melanoma cells by the three receptors of the β1 family. However, we found that Akt2 did not exhibit an inhibitory effect on the invasion of melanoma cells. In addition, in the cited report [33], the total activity of the β1-family was studied without determining the contribution of an individual receptor.

In conclusion, the results of this study can be considered in terms of clinical application. The mechanisms of almost all currently available targeted antitumor drugs are based on suppressing the activity of individual members of the signaling cascades maintaining the transformed phenotypes. Therefore, identification of previously unknown functions of signaling protein kinases is important for cancer therapy. Recently, clinical trials of several pan-Akt inhibitors in breast cancer are underway [35, 36]. Due to the pronounced side effects of pan-Akt inhibitors, Akt-isoforms specific inhibitors turned out to be promising. One of the allosteric inhibitors of Akt1 and Akt2 has been shown to be highly effective in suppressing tumor growth in mice and demonstrated moderate and transient side effects [29]. Obviously, antitumor effects of Akt isoforms are based on their canonical properties. However, if any neoplasms contain Akt isoforms with non-canonical traits, their use as a target in anticancer therapy may be harmful. Thus, it is important to take into consideration all individual functions of an anticancer target in a specific type of cancer.

## MATERIALS AND METHODS

### Cell culture and chemicals

SK-Mel-147 human melanoma line was obtained from the Memorial Sloan Kettering Cancer Center (USA). Cells were cultured in DMEM medium containing 10% fetal calf serum, 2 mM L-glutamine, 100 U/ml penicillin, and 100 μg/ml streptomycin and incubated at 37° C in an atmosphere with 5% CO_2_. Polyclonal antibodies against α3- and α5-integrin subunits were obtained from Chemicon (USA). Polyclonal antibodies against protein kinases Akt and Erk and their phosphorylated forms (pAkt Ser473 and pErk Thr202/Tyr204), protein kinase pFAK Tyr397, and proteins p53 and p21 were from Cell Signaling Tech (USA). Polyclonal antibodies to MMP-2 collagenase were from Santa Cruz Biothech (USA). Akt1- and Akt2-specific inhibitors, XXIII and XII, respectively, were purchased from Calbiochem (USA), and other chemicals were from Sigma (USA) unless otherwise stated.

### Transduction of cells with shRNA

Bacterial glycerol clones containing lentiviral plasmid vector pLKO.1-puro with shRNA specific for the α3- and α5-integrin subunits and pLKO.1-puro lentiviral vector with scrambled shRNA (control) were purchased from Sigma. Lentivirus was produced in HEK293T cells by co-transfection with shRNA-containing or scrambled shRNA-containing vector with packing plasmids as described earlier [37]. Cells were transduced with lentivirus in the presence of polybrene (8 μg/ml) and selected with puromycin (1–2 μg/ml) for 4–6 days.

### Clonal activity

About 2000 cells were plated on a 1% methylcellulose gel in complete medium in Petri dishes for 14 days. The colonies were stained with Crystal Violet, visualized in an optical microscope, and scanned.

### Invasion *in vitro*

The assays were performed as previously described [21]. Briefly, cells (5–7)·10^4^ in 150 μl DMEM containing 0.5% fetal serum were applied on Matrigel (BD Pharmingen, USA) immobilized on 8 μm-pore membranes of the upper chamber of Transwell clusters (Corning, USA) with the bottom chambers containing 1 ml of the same medium. After incubation for 48 h at 37° C, the medium was removed from the upper chamber, and cells from the upper surface of the membrane were carefully removed using cotton swabs. The chamber was incubated in a trypsin / versene solution, the cells migrated through the membrane were scrapped off, combined with cells that entered the trypsin / versene solution, centrifuged, suspended and counted in a cytometer.

### Anchorage-dependent apoptosis (anoikis)

Anoikis was assessed by the accumulation of cells non-survived after their cultivation on the non-adhesive substrate - polyhydroxyethylmethacrylate (poly-HEMA). The substrate was prepared in 6-well plates according to previously described procedure [38]. 3 x 10^5^ cells/well were incubated in medium containing 10% fetal serum, at 37° C for 24 h. Anoikis was assessed by percentage of cells stained *in vivo* with Trypan Blue, which was determined in an automatic cell analyzer Vi-Cell (Beckman Coulter).

### SDS-PAGE and western blotting

The procedures were performed as described in [38]. 30 μg of cell lysate proteins were run on SDA-PAGE and electroblotted onto a PVDF membrane. After reaction with specific primary antibodies, the membrane was incubated with HRP-conjugated secondary antibodies, developed in an ECL detection system (Amersham, England) and imaged on ChemiDoc (Bio-Rad). Quantification of protein levels from western blot analysis was done using Image Lab software (Bio-Rad).

### Statistical analysis

Differences between the groups were assessed using Student’s *t-*test and considered significant at *p* < 0.05.
